# Supplementary material for: Hi-BEHRT: Hierarchical Transformer-Based Model for Accurate Prediction of Clinical Events Using Multimodal Longitudinal Electronic Health Records
Source: IEEE J Biomed Health Inform. Author manuscript; Available in PMC 2023 Sep 12. (PMC7615082; doi:10.1109/JBHI.2022.3224727)
Supplement: Appendix [file EMS177288-supplement-Appendix.pdf]

Appendix

A. Additional Information on Dataset

In this section, we provide more information on modalities that are not commonly included in the modelling. More specifically, we will introduce procedure and test.

1) Procedure: Procedure is CPRD linked data collected from Hospital Episode Statistics (HES) Admitted Patient Care (EHS APC) data. It is recorded at the point of admission to, or attendances at NHS healthcare providers. All procedure information is coded using the U.K. Office of Population, Census and Surveys classification (OPCS) 4.6, and procedures that are not covered by OPCS code is not included in the system. Each record in the system is specified with a start and an end date, as well as event date. We used OPCS code and event date to structure the timeline of a patient's EHR history for modelling.

2) Test: Test is recorded in the CPRD test table and coded as Read code. It includes information on history/symptoms, examination/signs, diagnostic procedures, and laboratory procedures. In the experiment, we only used the information in the Read code level, which represents what examinations or procedures are carried out. More detailed quantitative information was excluded.

B. Clinical Codes for HF, Diabetes, CKD, and Stroke

TABLE V
ICD-10 CODES USED TO IDENTIFY PATIENTS WITH HEART FAILURE IN HOSPITAL DISCHARGE RECORDS AND GENERAL PRACTICE RECORDS

ICD Code	Description
I09.9	Rheumatic heart failure
I11.0	Hypertensive heart disease with (congestive) heart failure
I13.0	Hypertensive heart and renal disease with (congestive) heart failure
I13.2	Hypertensive heart and renal disease with both (congestive) heart failure and renal failure
I25.5	Ischemic cardiomyopathy
I27.9	Chronic cor pulmonale
I38	Congestive heart failure due to valvular disease
I42.0	Congestive cardiomyopathy
I42.1	Obstructive hypertrophic cardiomyopathy
I42.2	Nonobstructive hypertrophic cardiomyopathy
I42.6	Alcoholic cardiomyopathy
I42.8	Other cardiomyopathies
I42.9	Cardiomyopathy NOS
I50.0	Congestive heart failure
I50.1	Left ventricular failure
I50.2	Systolic (congestive) heart failure
I50.3	Diastolic (congestive) heart failure
I50.8	Other heart failure
I50.9	Cardiac, heart or myocardial failure NOS

I38 is mapped from Read code G580400

TABLE VI
ICD-10 CODES USED TO IDENTIFY PATIENTS WITH DIABETES IN HOSPITAL DISCHARGE RECORDS AND GENERAL PRACTICE RECORDS

ICD Code	Description
E10	Type 1 diabetes mellitus
E11	Type 2 diabetes mellitus
E12	Malnutrition-related diabetes mellitus
E13	Other specified diabetes mellitus
E14	Unspecified diabetes mellitus
O24.2	Pre-existing malnutrition-related diabetes mellitus

C. Model Evaluation Stratified By Baseline Age

We evaluated model performance stratified by the baseline age. The comparison was conducted on three subgroups of patients: 1) patients with baseline age between 35 and 50 years old (young adult); 2) patients with baseline age between 50 and 70 years old (middle-aged adult), and 3) patients with baseline age 70–90 years old (older adult). Table IX shows that the hierarchical BEHRT model has better performance across all subgroups, and it substantially outperforms for BEHRT model on HF and diabetes risk prediction tasks, especially for patients with younger age.

TABLE VII
ICD-10 CODES USED TO IDENTIFY PATIENTS WITH CKD IN HOSPITAL DISCHARGE RECORDS AND GENERAL PRACTICE RECORDS

ICD Code	Description
N18.1	Chronic kidney disease, stage 1
N18.2	Chronic kidney disease, stage 2
N18.3	Chronic kidney disease, stage 3
N18.4	Chronic kidney disease, stage 4
N18.5	Chronic kidney disease, stage 5
N18.9	Chronic kidney disease, unspecified
T86.1	Kidney transplant failure and rejection
I12.0	Hypertensive renal failure
N00	Acute nephritic syndrome
N03	Chronic nephritic syndrome
N04	Nephrotic syndrome
N05	Unspecified nephritic syndrome
N11	Chronic tubulo-interstitial nephritis
N13	Obstructive and reflux uropathy
N17	Acute renal failure
N19	Unspecified kidney failure
E10.2	Type 1 diabetes mellitus with kidney complications
E11.2	Type 2 diabetes mellitus with kidney complications

TABLE VIII
ICD-10 CODES USED TO IDENTIFY PATIENTS WITH STROKE IN HOSPITAL DISCHARGE RECORDS AND GENERAL PRACTICE RECORDS

ICD Code	Description
I60	Subarachnoid haemorrhage
I61	Intracerebral haemorrhage
I62	Other nontraumatic intracranial haemorrhage
I63	Cerebral infarction
I64	Stroke, not specified as haemorrhage or infarction
I65	Occlusion and stenosis of precerebral arteries, not resulting in cerebral infarction
I66	Occlusion and stenosis of cerebral arteries, not resulting in cerebral infarction
I67	Other cerebrovascular diseases
I68	Cerebrovascular disorders in diseases classified elsewhere
I69	Sequelae of cerebrovascular disease
G45.9	Transient cerebral ischaemic attack, unspecified
G46	Vascular syndromes of brain in cerebrovascular diseases

D. Size and Overlap of Sliding Window

For Hi-BEHRT model, we used sliding window to segment the raw EHR into segments. As shown in Table X when window size is relatively small (i.e., 50), the size of the stride does not have significant impact in terms of predictive performance, and the bigger stride size can potentially decrease the number of segments and reduce model complexity. However, for the larger window size (i.e., 100), the stride size becomes more important, and some level of overlap between segments is necessary. Without any overlap for window size 100, the AUPRC decreases 4% comparing to the model with stride size 50. Additionally, the analysis shows that not larger window size always the better choice. For instance, AUPRC of window size 100 without overlap decreases 2% comparing to AURPC of window size 50 without overlap. Without overlap, larger window can lead to shorter length in the segment level, and a balance between window size and length of segment might be more preferred in the hierarchical structure.

TABLE IX
BASELINE AGE STRATIFIED SUBGROUP ANALYSIS

			BEHRT		Hi-BEHRT	
Sample size	No. (%) of positive cases	Baseline age	AUROC	AUPRC	AUROC	AUPRC
HF						
154,032	1,008 (0.7)	35-50	0.84	0.40	0.90	0.56
180,416	6,878 (3.8)	50-70	0.88	0.64	0.93	0.72
111,044	17,670 (15.9)	70-90	0.86	0.75	0.90	0.80
Diabetes						
149,308	4,554 (3.1)	35-50	0.87	0.60	0.92	0.69
167,753	12,443 (7.4)	50-70	0.87	0.69	0.91	0.76
103,866	7,932 (7.6)	70-90	0.89	0.69	0.90	0.75
CKD						
145,889	4,343 (3.0)	35-50	0.88	0.62	0.89	0.64
176,422	13,037 (7.4)	50-70	0.90	0.74	0.92	0.76
111,727	24,875 (22.3)	70-90	0.89	0.83	0.91	0.84
Stroke						
136,090	11,325 (8.3)	35-50	0.88	0.70	0.88	0.71
157,789	21,392 (13.6)	50-70	0.88	0.76	0.90	0.79
93,159	22,793 (24.5)	70-90	0.87	0.82	0.89	0.84

TABLE X
PERFORMANCE OF HF RISK PREDICTION WITH DIFFERENT WINDOW AND STRIDE SIZE

Window size	stride size	AUROC	AUPRC
50	30	0.96	0.77
50	50	0.95	0.76
100	50	0.96	0.78
100	100	0.95	0.74
150	150	0.95	0.74

TABLE XI
HI-BEHRT HYPER-PARAMETER TUNING

Hidden size	Intermediate size	AUROC	AUPRC
150	108	0.96	0.77
90	108	0.95	0.74
240	108	0.96	0.77
150	256	0.96	0.77

E. Hyper-Parameter Tuning

We set up hierarchical BEHRT with similar hyper-parameters as the BEHRT model and used it as a reference model to tune the hidden size and intermediate size of the Transformer. More specifically, we applied grid search for hidden size among [90, 150, 240] and intermediate size among [108, 256]. All experiments were conducted on the 5-year HF risk prediction task. Table XI shows that hidden size 150 and intermediate size 108 can achieve similar performance as the model with larger size.

F. Evaluation for Multiple Levels of Hierarchy

In this section, we investigated how the number of levels of hierarchy in Hi-BEHRT can influence the model performance in risk prediction. Specifically, we compared the performance of Hi-BEHRT with two and three levels of hierarchy. This is because each additional level can substantially reduce the sequence length. For instance, a sequence with maximum length 1225 would reduce to sequence length 118 with window size 50 and stride size 10 after the first level of hierarchy and would further reduce to 7 after the second level of hierarchy. Therefore, our dataset limited the number of levels we can investigate, and it would not make sense to investigate Hi-BEHRT with more than three levels of hierarchy. We encourage future work to replicate our work to more comprehensively investigate Hi-BEHRT with more levels of hierarchy. In our experiment, we only modified the feature extractor and kept the total number of layers in feature extractor the same for both comparators. More specifically, the two-level Hi-BEHRT had one level of hierarchy with four layers of Transformer for the extractor while the three-level Hi-BEHRT included two levels of hierarchy with a two-layer Transformer for each hierarchy. Both comparators used window size 50 and stride size 10 and the rest parameters were the same as reported in the manuscript. The results show that both models achieved AUROC 0.96 and AUPRC 0.76 for HF risk prediction, and there is no material difference between two-level and three-level Hi-BEHRT in our dataset.
